# PPARα mediates night neon light-induced weight gain: role of lipid homeostasis

**DOI:** 10.7150/thno.50953

**Published:** 2020-09-15

**Authors:** Yishuang Luo, Julin Yang, Jinyu Kang, Kuihao Chen, Xiaofeng Jin, Frank J Gonzalez, Aiming Liu

**Affiliations:** 1 Medical School of Ningbo University, Ningbo 315211, China.; 2 Ningbo College of Health Sciences, Ningbo 315100, China.; 3 Laboratory of Metabolism, National Cancer Institute, NIH, Bethesda, MD 20892, USA.

**Keywords:** PPARα, weight gain, neon light, lipid homeostasis, circadian rhythm

## Abstract

**Rationale:** Light pollution leads to high risk of obesity but the underlying mechanism is not known except for the influence of altered circadian rhythm. Peroxisome proliferator-activated receptor α (PPARα) regulates lipid metabolism, but its role in circadian-related obesity is not clear.

**Methods:** Wild-type (WT) and *Ppara-*null (KO) mice on a high-fat diet (HFD) were treated with neon light at night for 6 weeks. Body weights were recorded and diet consumption measured. The hypothalamus, liver, adipose and serum were collected for mechanism experimentation.

**Results:** WT mice on a HFD and exposed to night neon light gained about 19% body weight more than the WT control mice without light exposure and KO control mice on a HFD and exposed to night neon light. The increase in adipose tissue weight and adipocyte size led to the differences in body weights. Biochemical analysis suggested increased hepatic lipid accumulated and increased transport of lipid from the liver to peripheral tissues in the WT mice that gained weight under neon light exposure. Unlike KO mice, the expression of genes involved in lipid metabolism and the circadian factor circadian locomotor output cycles kaput (CLOCK) in both liver and adipose tissues were elevated in WT mice under neon light exposure.

**Conclusions:** PPARα mediated weight gain of HFD-treated mice exposed to night neon light. More lipids were synthesized in the liver and transported to peripheral tissue leading to adaptive metabolism and lipid deposition in the adipose tissue. These data revealed an important mechanism of obesity induced by artificial light pollution where PPARα was implicated.

## Introduction

The global prevalence of obesity almost tripled between 1975 and 2016 in a report by World Health Organization, where 39% of adults were overweight and another 13% were obese in 2016 1. In the United States, the obesity rate was obviously higher than those in other developed countries, with more than 1/3 of adults being affected 2. By 2030, 90% of American adults will be overweight and 51% will be obese 3. In developing countries such as India, more than 135 million people were affected by obesity in 2018 4. In China, the population of obese people became the highest in the world in 2016 5.

Obesity is a major risk for dyslipidemia, diabetes, hypertension, cancer and musculoskeletal disorder 6. Adipokine production and chronic inflammation drove metabolic dyslipidemia and accelerated the development of cardiovascular diseases in obese patients 7, 8. Obesity was also an important contributing factor to type 2 diabetes mellitus (T2DM). In children and teenagers, severe obesity was reported to increase the risk of T2DM compared with that in adults 9. Persistent myocardial lipid toxicity in obese type 2 diabetics was more likely to cause cardiac systolic dysfunction than in non-obese patients 10. In addition, obese people were 3.5 times more likely to develop high blood pressure 11. In another report, the prevalence of hypertension was much higher in obese individuals compared with lean individuals (42.5% *vs.* 15.3%) 12.

Exposure to light is known to result in many systemic diseases. Artificial light affected the production of hormones which disrupted the endocrine balance. This was supposed to be the reason why night work led to an increased risk of breast cancer 13. A more serious metabolic disorder associated with exposure to artificial light at night was obesity. Compared to darkness, sleeping with the TV or lights on increased body weight by 5 kg and body mass index (BMI) by 10% 14. In shift workers, the increase of BMI was significantly higher than that of day workers according to a 14-year study 15, 16. Countercyclical work and night light exposure disrupted the rhythms of metabolism, resulting in an excess of calorie stores and weight gain 17, 18. However the mechanism underlying fast weight gain caused by night light exposure is not known.

Peroxisome proliferator-activated receptor α (PPARα) is involved in metabolism, transport, binding and uptake of lipids 19, 20. In mice deficient with hepatic PPARα, a 12-week high-fat diet (HFD) triggered development of liver inflammation and elevated plasma total cholesterol (TC) 21. In mice challenged with a diet supplemented with hydrogenated coconut oil, a two-fold increase in insulin and a modest increase in glucose were observed. However, mice lacking functional PPARα maintained normal insulin levels and were protected from hyperinsulinemia 22. In control *Ppara^fl/fl^* and the hepatocyte specific PPARα knockout mice (*Ppara*^ΔHep^) mice, 12-week HFD feeding increased the body weight, and no difference between them was observed 21. In an aging model, hepatic lipid accumulation and hypercholesterolemia were only observed in *Ppara*^ΔHep^ mice 23, 24. Thus, the hepatic PPARα plays a critical role in lipid metabolism, but the effect might be protective or disruptive, depending on the models. Nevertheless, in weight gain of mice exposed to artificial light, the role of PPARα is not clear.

Reciprocal regulation between PPARα and the circadian clock was reported 25. Circadian locomotor output cycles kaput (CLOCK) and brain and muscle ARNT-like 1 (BMAL1) are central elements of the circadian regulatory network. In CLOCK-mutant mice, the diurnal expression PPARα in the liver disappeared 26, 27. In *Bmal1*^-/-^ mice, the expression of PPARα in the liver was significantly down-regulated 28. In turn, PPARα was reported to regulate the transcription of the circadian factors 29, 30. In the liver of *Ppara*-null mice, the amplitude of *Bmal1* decreased and that of *Per3* increased. In regulation of the hepatic *Bmal1* expression, PPARα bound a peroxisome proliferator-activated receptor element on the *Bmal1* promoter 28. Additionally, activation of PPARα by bezafibrate was observed to stimulate the circadian expression of *Per2* in peripheral tissues 29. However the mechanism by which crosstalk between CLOCK/BMAL1 and PPARα disrupts metabolism remains to be explored.

In this study, wild-type (WT) and *Ppara*-null (KO) mice on HFD were exposed to neon light at night. Lipid metabolism and circadian rhythms were investigated to explore the role of PPARα in weight-gain of mice exposed to neon light at night that mimicks artificial light pollution in city life.

## Methods

### Materials and reagents

High-fat diet (HFD; 45% fat, 35.6% carbohydrate, 19.4% protein) and normal diet (ND; 10% fat, 75.9% carbohydrate, 14.1% protein) were obtained from Nantong Trophy Feed Technology (Nantong, China). The neon lights were bought from Hengyida Lighting (Changzhou, China). Kits for the detection of total cholesterol (TC), triglyceride (TG) and low density lipoprotein cholesterol (LDL-C) were acquired from Meikang Biotechnology (Ningbo, China). The ELISA kit for apolipoprotein B (ApoB) was from DEVELOP (Wuxi, China). TRIzol was purchased from Omega Bio-Tek (Guangzhou, China). The kit for reverse transcription and UltraSYBR Mixture were purchased from CWBIO (Beijing, China). The BCA kit for protein quantification and 5X SDS-PAGE loading buffer were obtained from Beyotime Biotechnology (Shanghai, China). The antibody against CLOCK was purchased from Signalway Antibody (SAB, USA). Antibodies against APOB (1:1000, ab20737), BMAL1 (1:5000, ab93806) and GAPDH (1:5000, ab181602) were bought from Abcam (MA, USA). Ready-for-use hematoxylin-eosin and those agents for western blot were bought from Solarbio (Shanghai, China).

### Animals and treatments

The *Ppara*-null (KO) mice were described in earlier studies (PMID: 753910) and were on the 129/Sv genetic background as were the WT control mice. Mice were treated in accordance with the Institute of Laboratory Animal Resource guidelines under protocols approved by the Animal Care and Use Committee of Ningbo University. The housing environment was 24 ± 1 °C, with a humidity of 50-70% and a 12-h light-dark cycle.

Both the WT and KO mice were randomly assigned to 4 groups: WT-LD-ND and KO-LD-ND (12-h light-dark cycle, normal diet, n = 8); WT-LL-ND and KO-LL-ND (12-h light-light cycle, normal diet, n = 8); WT-LD-HFD and KO-LD-HFD (12-h light-dark cycle, HFD, n = 8); WT-LL-HFD and KO-LL-HFD (12-h light-light cycle, HFD, n = 8). Daytime lighting was the same for all groups. Neon light exposure around 70 lux was provided to groups of 12-h light-light cycle from Zeitgeber 12 (ZT12, 8:30 pm) to Zeitgeber 0 (ZT0, 8:30 am). The mice were scaled every week and the food intake was measured every two days. After treatment for 6 weeks, all the mice were sacrificed at ZT12 (n = 4) and ZT0 (n = 4) respectively. The serum was stored at -20 °C and tissues (hypothalami, livers and subcutaneous adipose tissues in the groin) collected were flash frozen on dry ice and kept in -80 °C refrigerator.

### Histopathology analysis

The adipose tissues fixed in 10% formalin buffer were subject to gradient dehydration, transparency, waxdip and embedding. Serial 6 μm sections were cut from paraffin block, which were then stained with haematoxylin and eosin (HE stain). Finally, the sections were observed using microscope (Carl Zeiss, Axiostar plus). The size of fat cells was measured using Image J 1.8.0.

### Biochemical analysis

To measure the TC and TG in liver, 20 mg liver tissues were cut and 180 μL normal saline added. The samples were then homogenized at 6500 r/min for 30 s using MagNALyser (Roche, USA). The supernatant was extracted after centrifugation and the analysis procedures were performed according to the descriptions in the kits. The LDL-C level in the serum samples were determined by an enzymatic colorimetry using the Multiskan GO (Thermo, USA). The serum ApoB concentration was detected by ELISA following the instructions after 1:3 dilution.

### RNA quantification

Frozen liver and adipose tissues were lysed in TRIzol and then homogenized using the above MagNALyser. The RNA concentration was quantified and its purity was determined by OD260/OD280 value. Reverse transcription was performed the same as previously described 31. The primer sequences are listed in Table 1. Quantitative polymerase chain reaction (Q-PCR) amplification on 384-well plates was performed in a 5 μL system containing 1 μL cDNA, 2.2 μL UltraSYBR Mixture, 0.1 μL primer and 1.6 μL purifed water using LightCycler 480Ⅱ(Roche, USA). The 2 ^-ΔΔCt^ was used to quantify the expressions of target genes. Measured mRNA abundance was normalized to 18S rRNA and expressed as fold change equivalent to that of WT-LD-ND or KO-LD-ND.

### Western Blot assay

The frozen liver, adipose tissues and hypothalamic tissues were lysed in RIPA with 1% PMSF and then homogenized adequately. The samples were centrifuged at 13000 r/min for 20 minutes under 4 °C to collect the supernatant followed by protein quantification and normalization. An equal volume of 5X SDS-PAGE loading buffer was mixed with the samples, and then boiled for 8 min. Proteins in tissues were transferred to PVDF membranes for 1 h after separation using 10% SDS-polyacrylamide gels for 1.5 h. To assay the serum APOB, proteins in serum samples were separated for 2 h *via* 4% SDS-polyacrylamide gels and the transferring continued for 6 h. All membranes were blocked with 5% milk for 3.5 h. The membranes were incubated with primary antibody at 4 °C overnight. After washing with phosphate buffer, it was incubated with a secondary antibody for 2 h at room temperature. ECL substrates were added to the blotted PVDF and the images were recorded by chemiluminescence imaging system ChemiScope 6100 Touch (Shanghai, China). The densitometry analysis of the protein bands was done using Image J 1.8.0.

### Statistical analysis

The data were expressed as the mean ± SEM. Data analysis was performed using SPSS 17.0 for Windows. In expression of lipid synthesis and catabolism, the total transcription levels were averaged from the Q-PCR results of genes involved in each function. One-way ANOVA and Student's t test were carried out for difference examination. The analyzed data was plotted by GraphPad Prism 7.0. Significance was considered different when *P* values were below 0.05 which was indicated by asterisk.

## Results

### Weight gain in WT mice was stimulated by neon light exposure

**Figure 1:**
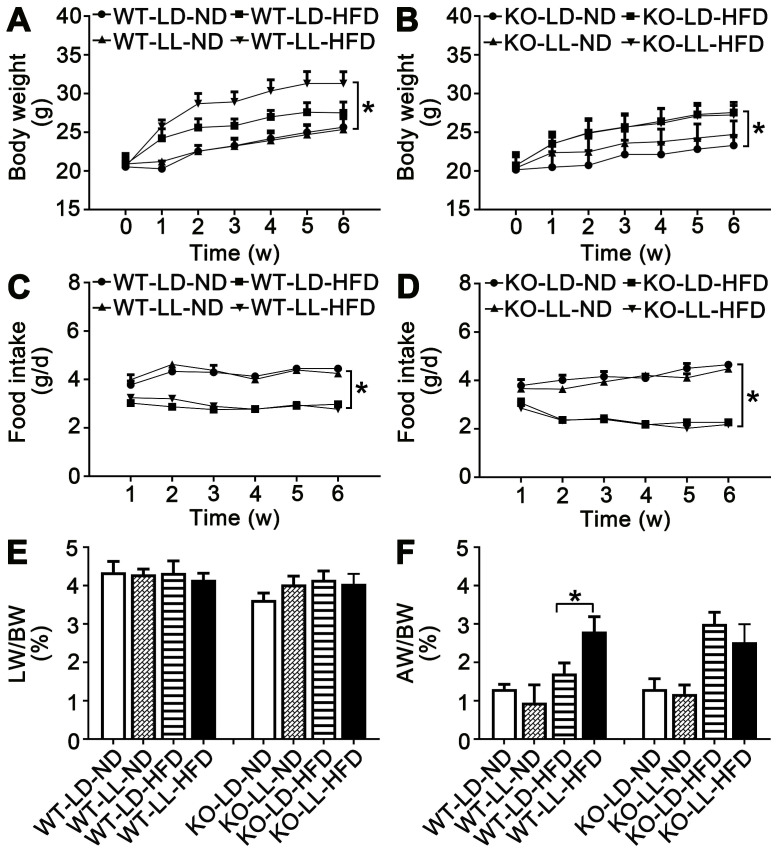
** Body weight gain of WT mice on HFD was stimulated by night neon light exposure.** (A-B) Body weight increase of the WT and KO mice over the course of the experiment. (C-D) Food consumption of the WT and KO mice during the experiment. Changes in the ratio of liver weight (E) and adipose weight (F) to body weight throughout the experiment. Data are presented as mean ± SEM (n = 8, *P < 0.05 compared with the LD-ND group).

The body weight of the mice in the 8 groups was comparable when the treatment began. The mice in group WT-LD-ND and WT-LL-ND gained 4.5-5.1 g of weight when the treatment finished while mice in group KO-LD-ND and KO-LL-ND gained 3.2-4.3 g. The weight gain of mice in WT-LL-HFD (*P =* 0.014) was much higher than that of WT-LD-HFD (10.8 g *vs.* 6.7 g) (Figure 1A). However, weight gain of mice in KO-LD-HFD (*P =* 0.047) and KO-LL-HFD was similar (6.8 g *vs.* 6.5 g) (Figure 1B). Thus, exposure to neon light stimulated weight gain in WT mice but not in KO mice and this occurred when the mice were on a HFD not normal diet.

During the treatment, the diet consumption of mice in the WT-LD-ND, WT-LL-ND, KO-LD-ND and KO-LL-ND groups was about 4 g/day. The food intake was around 3 g/day for mice in both the WT-LD-HFD and WT-LL-HFD groups (Figure 1C). In contrast, this amount of food consumed by the KO-LD-HFD and KO-LL-HFD groups was about 2.5 g/day (Figure 1D). HFD contributed to weight gain of both WT and KO mice compared with their control groups treated with normal diet, as expected. But the fast weight gain of the WT mice on HFD exposure to neon light was not related to the diet consumption.

There was no difference in the liver weight/body weight (LW/BW) among the 4 WT groups. For the KO mice, there was a slight increase when they were exposed to HFD and neon light (Figure 1E-F). Compared with WT-LD-ND, the adipose weight/body weight (AW/BW) of WT-LD-HFD was increased 0.3-fold and WT-LL-HFD was increased 1.2-fold (*P =* 0.002). For the KO mice, the AW/BW increase was 1.3- and 0.9-fold in the KO-LD-HFD and KO-LL-HFD groups respectively (Figure 1G-H). The tendency of the fat weight increase matched the body weight gain of the mice in all groups of two mouse lines.

**Figure 2:**
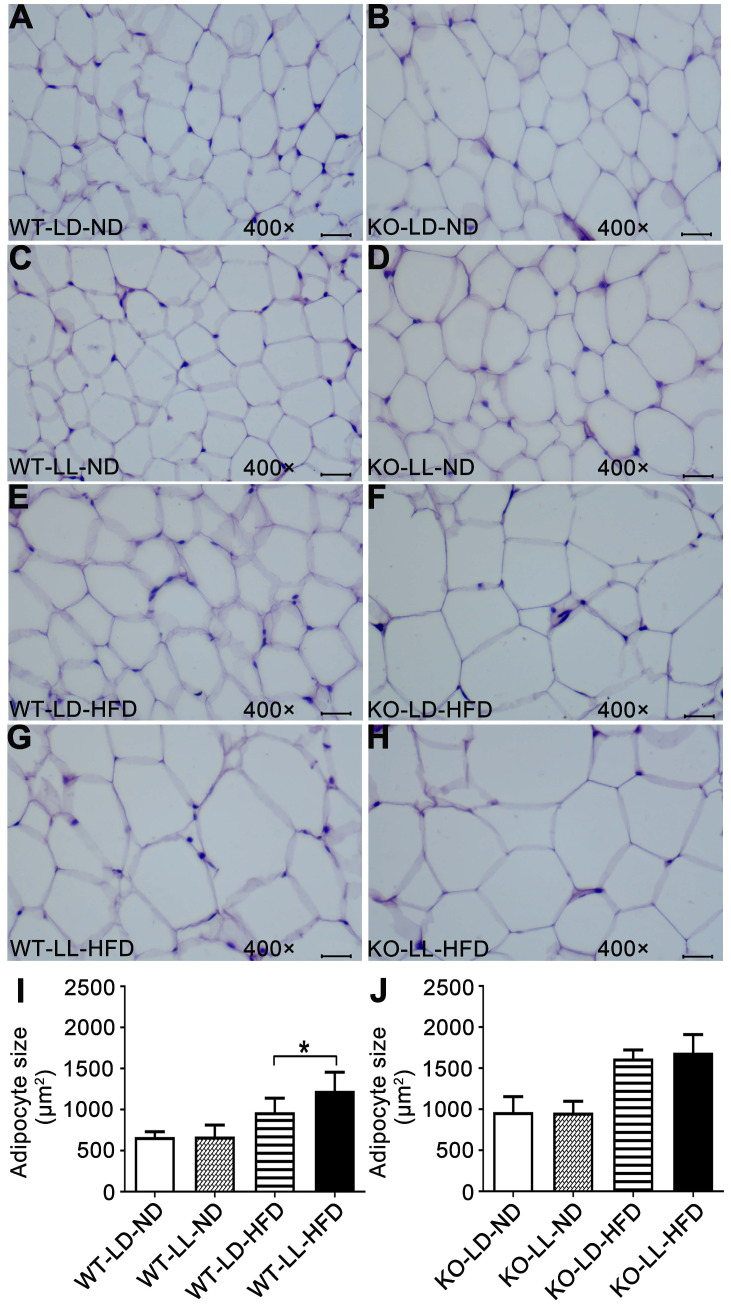
** Adipocyte enlargement correlated with the fast weight gain of WT mice.** (A-H) Histopathological analysis of adipose tissues (40×). Scale bar = 25 µm. (I-J) The size of fat cells in each group. Data are presented as mean ± SEM (n = 8, * meant *P* < 0.05).

Histopathology analysis of adipose tissue showed the adipocytes in WT-LL-ND group were of the same size as those in the WT-LD-ND group. In the WT-LD-HFD group, the size of adipocytes was increased by 0.5-fold (*P =* 0.017). But the size was increased by nearly 1-fold in the WT-LL-HFD group (*P =* 0.000). The size of adipocytes in KO mice on normal diet was almost the same as those of WT mice. For the mice in the KO-LD-HFD and KO-LL-HFD groups, the adipocyte size increase was both around 0.7-fold (Figure 2). This tendency of adipocyte enlargement was almost the same as the fat weight gain, as well as the body weight gain under the different treatments.

### Lipid homeostasis was disrupted in WT mice gaining most weight

**Figure 3:**
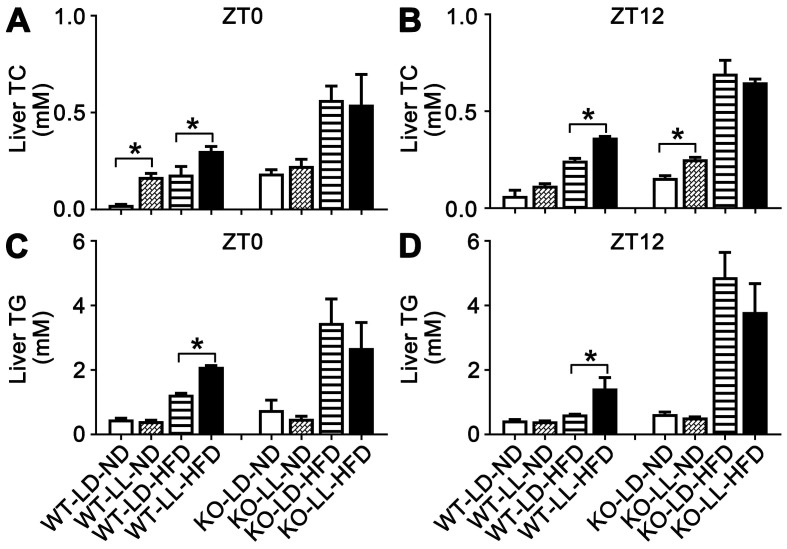
** The liver TC and TG accumulation in WT mice were increased by night neon light exposure.** (A-B) The levels of TC in livers at ZT0 and ZT12 in two mouse lines. (C-D) The levels of TG in livers at ZT0 and ZT12 in two mouse lines. Data are presented as mean ± SEM (n = 4, * meant* P* < 0.05). TC: total cholesterol; TG: triglyceride; ZT0: Zeitgeber 0; ZT12: Zeitgeber 12.

The liver TC in the WT-LL-ND and WT-LD-HFD groups were increased by 6.1- (*P =* 0.050) and 6.6-fold at ZT0. The TC in the WT-LL-HFD group was increased about 11.7-fold (*P =* 0.035) by light exposure. At ZT12, TC in the three experimental groups also increased but the difference between the WT-LL-HFD and WT-LD-HFD groups was lower than that at ZT0. TC in the KO-LL-ND group was at the same level as that in the control group at both time points. The increase of TC was around 2-fold at ZT0 and 3-fold at ZT12 in both the KO-LD-HFD and KO-LL-HFD groups compared with the control group. However, no difference between the two HFD treated KO groups was detected at ZT0 or ZT12 (Figure 3A-B).

For hepatic TG, the level in the WT-LD-HFD group was increased by 1.6-fold and in the WT-LL-HFD group was increased by 3.5-fold (*P =* 0.049) at ZT0, showing a big difference. At ZT12, the TG was also increased more in the WT-LL-HFD group mice than that in the WT-LD-HFD group mice (2.3-fold *vs.* 0.4-fold) (*P =* 0.033), where the difference was even sharper. In contrast in the KO mice, HFD treatment increased the TG group by 3.5- and 2.5-fold in the KO-LD-HFD and KO-LL-HFD groups, respectively at ZT0, compared with the control group. At ZT12, this increase was amplified to be 6.7-fold and 5.0-fold respectively. Again, no difference was found between the two KO groups, at either ZT0 or ZT12 (Figure 3C-D).

**Figure 4:**
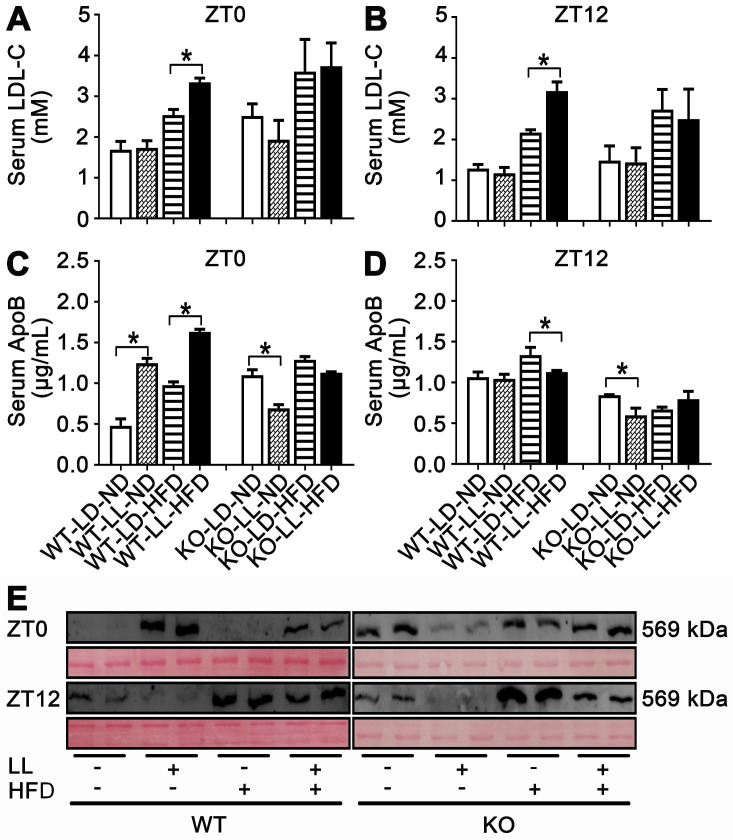
** Lipid transportation out of liver was increased in WT mice exposure to night neon light.** (A-B) Serum LDL-C concentrations at ZT0 and ZT12 in WT and KO mice. (C-D) ApoB concentrations in serum at at ZT0 and ZT12 in two mouse lines. Data are presented as mean ± SEM (n = 4, * meant* P* < 0.05). (E) Serum APOB protein levels at two time points (n = 2). ApoB: apolipoprotein B; LDL-C: low density lipoprotein cholesterol; ZT0: Zeitgeber 0; ZT12: Zeitgeber 12.

Serum LDL-C in the WT-LL-ND group was the same as that in the control group and the level in the WT-LD-HFD group was slightly increased by 0.5-fold. The increase in the WT-LL-HFD group was 1.0-fold (*P =* 0.044) compared with that of the WT-LD-ND group at ZT0. LDL-C levels were more pronounced in mice at ZT12, with a 0.7-fold increase in the WT-LD-HFD group and a 1.5-fold increase in the WT-LL-HFD group (*P =* 0.030). At ZT0, the LDL-C levels in the KO-LD-HFD and KO-LL-HFD groups were both increased by about 0.5-fold. At ZT12, the level of was increased by 0.8-fold in the KO-LD-HFD group, and increased by 0.7-fold in the KO-LL-HFD group (Figure 4A-B). Unlike the WT mice, LDL-C levels in group KO-LL-HFD group did not surpass that in the KO-LD-HFD group at either ZT0 or ZT12.

As carrier protein, serum ApoB levels were measured by two approaches. Combining the ELISA results and western-blot of serum sumples, it is clear that ApoB levels in the WT-LL-ND and WT-LL-HFD groups were higher than in the WT groups without neon light exposure at ZT0 (*P <* 0.05). In contrast, at ZT12, the ApoB levels in the WT-LL-ND and WT-LL-HFD groups were significantly lower than the WT groups without neon light exposure (*P <* 0.05). For the KO mice, whether at ZT0 or ZT12, the ApoB levels in the KO-LL-ND and KO-LL-HFD groups were lower than that in group KO-LD-ND and KO-LD-HFD groups (*P <* 0.05) (Figure 4C-E). These data suggested more lipid transport from liver to peripheral tissues in WT mice exposed to neon light than occured in KO mice.

### Lipid metabolism in WT mice were altered by neon light exposure

**Figure 5:**
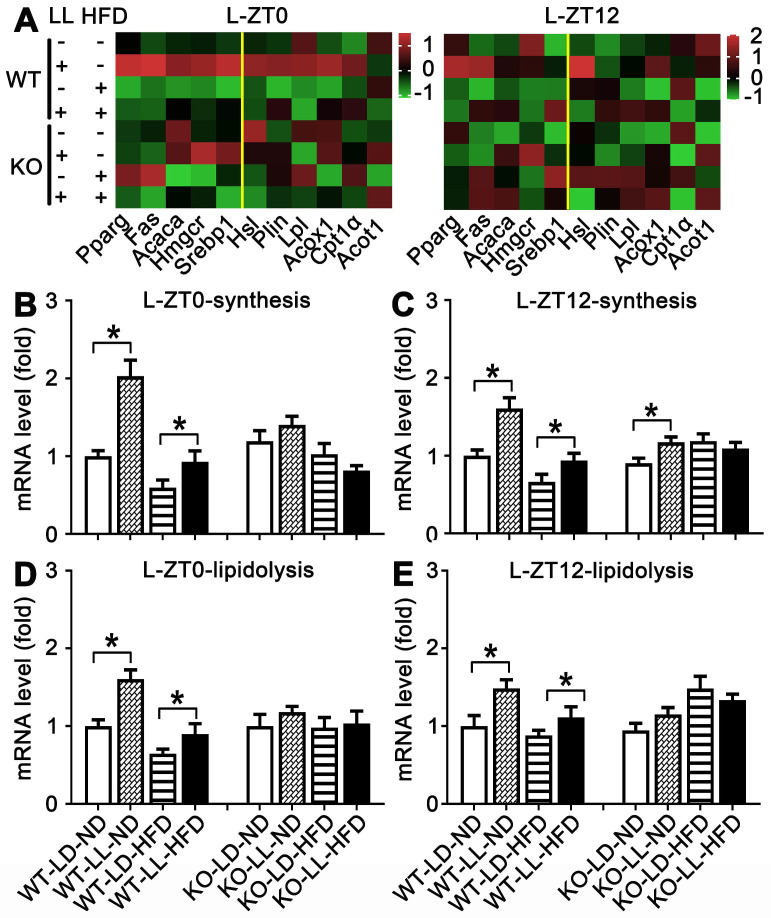
** Lipid metabolism rhythm in the liver in WT mice was enhanced by night neon light exposure.** (A) Metabolic gene expression heat map at ZT0 and ZT12. (B-C) The average transcription changes of metabolic gene related to lipid synthesis at ZT0 or ZT12. (D-E) The average transcription changes of metabolic gene related to lipidolysis at ZT0 or ZT12. Data are presented as mean ± SEM (n = 4, * meant* P* < 0.05). *Acaca*: acetyl-CoA carboxylase;* Acot1*: acyl-CoA thioesterase1; *Acox1*: acyl-CoA oxidase 1;* Cpt1a*: carnitine palmityl transferase 1;* Fas*: fatty acid synthetase;* Hmgcr1*: hydroxymethyl glutaryl coenzyme reductase 1; *Hsl*: hormone-sensitive lipase;* Lpl*: lipoprotein lipase;* Plin*: perilipin; *Pparg*: peroxisomal proliferator-activated receptor gamma; *Srebp1*: sterol regulatory element-binding protein 1; ZT0: Zeitgeber 0; ZT12: Zeitgeber 12.

**Figure 6:**
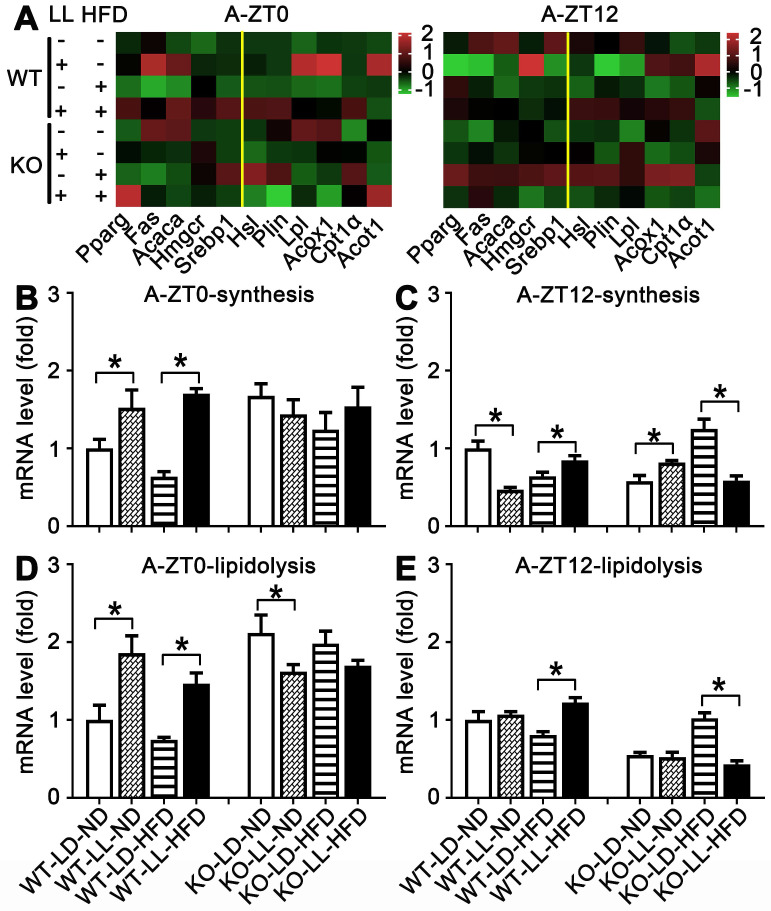
** Lipid metabolism rhythm in the adipose tissue was altered adaptively.** (A) Metabolic gene expression heat map at ZT0 and ZT12. (B-C) The average transcription changes of metabolic gene related to lipid synthesis at ZT0 or ZT12. (D-E) The average transcription changes of metabolic gene related to lipidolysis at ZT0 or ZT12. Data are presented as mean ± SEM (n = 4, * meant* P* < 0.05). *Acaca*: acetyl-CoA carboxylase;* Acot1*: acyl-CoA thioesterase1; *Acox1*: acyl-CoA oxidase 1;* Cpt1a*: carnitine palmityl transferase 1;* Fas*: fatty acid synthetase;* Hmgcr1*: hydroxymethyl glutaryl coenzyme reductase 1; *Hsl*: hormone-sensitive lipase;* Lpl*: lipoprotein lipase;* Plin*: perilipin; *Pparg*: peroxisomal proliferator-activated receptor gamma; *Srebp1*: sterol regulatory element-binding protein 1; ZT0: Zeitgeber 0; ZT12: Zeitgeber 12.

The mRNA expressions of 11 genes related to lipid synthesis and catabolism was analyzed in the livers and adipose tissues (Figures 5 & 6). In the livers of WT mice, the average expression levels of both synthesis-related genes and lipisolysis-related genes at ZT0 and ZT12 were up-regulated in the WT-LL-ND group (*P <* 0.05). Compared with the WT-LD-HFD group, expression of two groups of genes in the WT-LL-HFD group was also up-regulated at two time points (*P <* 0.05). Differently in the livers of KO mice, gene expressions in the KO-LL-ND and KO-LL-HFD groups were similar to their corresponding groups without light exposure whether at ZT0 or ZT12.

In the adipose tissue of WT mice, the average gene expression in both the WT-LL-ND and WT-LL-HFD groups were higher than their control counterparts (*P <* 0.05), except that synthesis-related genes in the WT-LL-ND group was the down regulated at ZT12 (*P =* 0.000). The expression of these genes in the KO-LL-ND and KO-LL-HFD groups at ZT0 were not modified. But they were down-regulated in KO mice on HFD exposed to neon light at ZT12 (*P <* 0.05), which was quite different from those in WT mice. These data from liver and adipose tissues suggested a circadian regulation of lipid metabolism mediated by PPARα when the mice were challenged with neon light exposure at night.

### CLOCK was involved in modification of peripheral circadian rhythm

**Figure 7:**
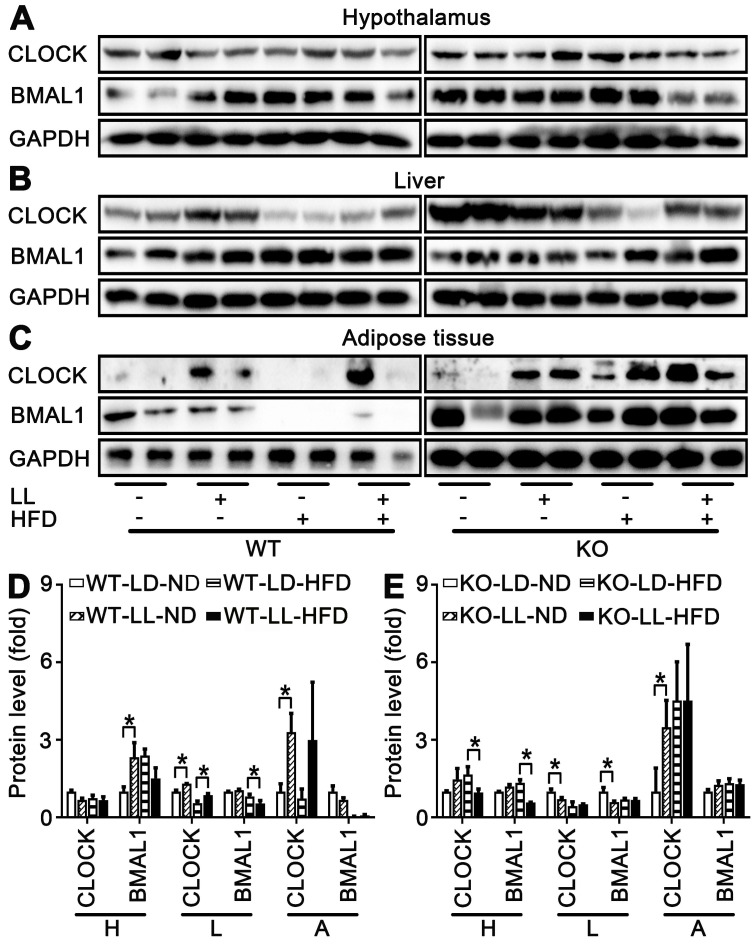
** Peripheral CLOCK was involved in disruption of lipid homeostasis induced by night neon light exposure.** Changes of core clock proteins CLOCK and BMAL1 in hypothalamus (A), liver (B) and adipose tissue (C). Densitometry changes in CLOCK expression in three tissues of WT mice (D) or KO mice (E). Data are presented as mean ± SEM (n = 2, * meant* P* < 0.05). BMAL1: brain and muscle ARNT-like 1; CLOCK: circadian locomotor output cycles kaput; H: hypothalamus; L: liver; A: adipose tissue; KO: *Pparα-null*; WT: wild-type.

Expression of circadian factors at ZT0 were analyzed as an example to exhibit their involvement in disruption of lipid homeostasis shown above. There were no significant changes in core circadian factor CLOCK and BMAL1 in the hypothalamus of either WT mice or KO mice. However, differences in their regulation by HFD or light exposure in the liver and fat tissues were quite obvious. In livers and adipose, compared with the control groups, CLOCK expression was significantly up-regulated in both the WT-LL-ND and WT-LL-HFD groups (*P <* 0.05). BMAL1 was not changed significantly in the WT-LL-ND or WT-LL-HFD groups compared with the corresponding WT groups without neon light exposure (Figure 7A-D). In the KO mice, the CLOCK expression in livers and adipose tissues were not modified (*P <* 0.05) except an increase in adipose tissues. Changes of BMAL1 in both livers and fats were also not significant in KO mice, similar as those in WT mice (Figure 7A-C, E). These data showed that the effect of circadian rhythm was amplified in the liver and adipose tissues in KO mice. Additionally, CLOCK was involved more in the disruptive action than BMAL1.

## Discussion

Obesity has becoming a public health problem over the past few decades. The reason behind this epidemic is lifestyle rather than genetic mutations 32. HFD treatment usually takes 8-20 weeks to produce a 6-10 g weight gain 33, 34. Under 10-h dim night light for 4 weeks, the mice gained more weight and daytime food intake was increased by 10% 35. The body weight of mice exposed to 8-h dim light for 6 weeks showed the same tendancy as the above, but the food intake was reduced by 2 g/week 36. In this study, phenotype was highly significant when the mice were treated with HFD for only 6 weeks. Both WT and KO mice gained more weight on HFD compared those on normal diet. Interestingly, under exposure to neon light, the WT mice gained 19% more weight than the KO mice when both were treated with HFD. Additionally, HFD consumption was not different between two mouse lines during the 6 weeks. These data suggested the weight gain was accelerated when mice were challenged with both light exposure and HFD. This effect was related to the modification of metabolism, not diet consumption.

Visceral adipose tissues and subcutaneous adipose tissues are different in structure and function. The former are around the abdominal viscera and the latter are at subcutaneous areas 37. Excess free fatty acids and TGs are stored in the fat cells of the subcutaneous adipose tissues, which was investigated in obesity 38. In this study, the tendency of subcutaneous adipose weight correlated well with the changes of body weight in each group. It was suggested that the weight increase of fat tissues contributed mostly to body weight gain of mice under night light exposure. Specifically, the size of adipocytes was increased significantly, which closely related with fat weight gain. So, the accumulation of excess lipids drove the enlargement of fat cells, and then the body weight gain of the mice challenged with HFD and night light exposure.

In mice with non-alcoholic steatohepatitis induced by HFD treatment for 10 weeks, the liver TG was increased by 3-fold, and the increase of TG and LDL-C in serum was about 1-fold 39. Also in obese mice treated with HFD, liver TG increase was observed, but TG in the plasma was decreased 21. In this study, TC and TG levels in the liver were both significantly increased, suggesting lipid was over-produced in the liver. Both biomarkers were increased most in the WT mice under light exposure and HFD. These modifications did not occur in KO mice on HFD, regardless of night light exposure. Additionally, LDL-C and ApoB levels in serum were consistent with those of TG and TC in the liver between two mouse lines or different diets. Thus the lipid transport to the adipose tissues *via* ApoB was more active in mice challenged with light exposure and HFD, leading to an adaptive metabolism in adipocytes. Since the strong involvement of PPARα in lipid metabolism, some of the biomarkers between WT and KO countergroups were quite different, so they were not directly compared. The different tendencies of these biomarkers between WT and KO mice were sufficient enough to suggest PPARα's role in mediating the disruption of lipid homeostasis in WT mice.

The expression of genes related to fat metabolism was not correctly induced or repressed in *Pparα*^Hep-/-^ mice, which impaired fatty acid catabolism. And more severe lipid metabolism disorders were occurred in *Pparα*^-/-^ mice. Blood sugar and body temperature were lower in mice with *Pparα*^Hep-/-^ rather than *Pparα*^-/-^ mice under fasting, indicating the PPARα activity in non-hepatic tissues 23. Non-hepatic PPARα reduced systemic lipid load by elevating fatty acid oxidation and lipase activity when hepatic lipid metabolism was impaired, suggesting the adaptation of extrahepatic PPARα's role 40. However, when lipids were overloaded, the liver responded firstly and release hepatogenic vesicles which move to adipose cells to regulate adipogenesis 41. Combinine these reports and the data in this work, it was suggested that the hepatic PPARα in lipid metabolism was predominant and those in non-hepatic tissues were adaptative.

Besides PPARα, its target genes related to the lipid metabolism were also reported to be regulated by the biological clock 42. In this experiment, diurnal variations in gene expressions of WT mice were more active in the liver at ZT0/12 and in the adipose tissues at ZT0 under light exposure. However, they were decreased in the HFD-treated mice. PPARα target gene expression in adipose tissue of WT mice were significantly down-regulated in the two groups exposed to neon light at ZT12, especially in the group with light exposure only. But the difference of gene expressions in the KO groups was weaker and irregular compared with those in WT mice. The combinative effect of these alterations was more synthesis of lipid in the liver and more transportation of lipid to the adipose tissues. This is a potential mechanism of the increased weight gain in WT mice exposed to night light.

The central clock suprachiasmatic nucleus located in the hypothalamus coordinates oscillators in peripheral tissues to keep normal circadian rhythms 26, 43. The peripheral circadian rhythms can be modified by diet composition and ingestion behavior 44. In a mouse model, the liver, kidney and lung clocks were advanced by a 2-week treatment of high-salt diet 45. In this study, the expression of CLOCK and BMAL1 in the hypothalamus were not modified. But in the liver and adipose tissues, CLOCK was modified by both neon light exposure in WT mice on both HFD and normal diet. Therefore, it was reasonable to suppose that CLOCK was involved more in crosstalk with PPARα under the neon light exposure at night. However, which was upstream or downstream between CLOCK and PPARα in retulating lipid homeostasis remains to be investigated, considering their reported reciprocal regulation.

Brown adipose tissues promote energy metabolism and produces heat. Metabolic syndrome in mice on a HFD could be reduced by exosomes derived from brown adipose tissue 46. White adipose beiging was reported to be involved in improvement of energy metabolism. Thus there is some chance that brown adipose tissues and white adipose tissues beiging might be regulated in this work. Additionally, there was a black box in this work indicated in the graphical abstract. Since the regulation between PPARα and the circadian clock was reciprocal, it was difficult to conclude which one was the up-stream of the other in the development of obesity induced by night light pollution 25.

In this study, WT mice on a HFD exposed to night neon light pollution gained more weight in comparison with the KO mice, suggesting the critical role of PPARα. More lipids was synthesized in the liver and then transported to the adipose tissues where adaptive responses drove adipocyte enlargement, potentiated by crosstalk between CLOCK and PPARα. These data revealed an important mechanism of obesity induced by artificial light pollution where the etiological role of PPARα was suggested.

## Figures and Tables

**Table 1:** The primers used for the Q-PCR assessment in this study

Genes	Forward primer	Reverse primer	Size (bp)
*Pparg*	GGAAGACCACTCGCATTCCTT	GTAATCAGCAACCATTGGGTCA	121
*Fas*	TATCAAGGAGGCCCATTTTGC	TGTTTCCACTTCTAAACCATGCT	95
*Acaca*	GATGAACCATCTCCGTTGGC	GACCCAATTATGAATCGGGAGTG	65
*Hmgcr1*	AGAGCGAGTGCATTAGCAAAG	GATTGCCATTCCACGAGCTAT	84
*Srebp1*	TGGTTGTTGATGAGCTGGAG	GGCTCTGGAACAGACACTGG	96
*Hsl*	GATTTACGCACGATGACACAGT	ACCTGCAAAGACATTAGACAGC	114
*Plin*	CAAGCACCTCTGACAAGGTTC	GTTGGCGGCATATTCTGCTG	92
*Lpl*	TTGCCCTAAGGACCCCTGAA	TTGAAGTGGCAGTTAGACACAG	88
*Acox1*	CCGCCACCTTCAATCCAGAG	CAAGTTCTCGATTTCTCGACGG	86
*Cpt1α*	TGGCATCATCACTGGTGTGTT	GTCTAGGGTCCGATTGATCTTTG	133
*Acot1*	TGGTTGTTGATGAGCTGGAG	GGCTCTAACCGTATG	107
*18S rRNA*	ATTGGAGCTGGAATTACCGC	CGGCTACCACATCCAAGGAA	102

Acaca: acetyl-CoA carboxylase; Acot1: acyl-CoA thioesterase1; Acox1: acyl-CoA oxidase 1; Cpt1a: carnitine palmityl transferase 1; Fas: fatty acid synthetase; Hmgcr1: hydroxymethyl glutaryl coenzyme reductase 1; Hsl: hormone-sensitive lipase; Lpl: lipoprotein lipase; Plin: perilipin; Pparg: peroxisomal proliferator-activated receptor gamma; Srebp1: sterol regulatory element-binding protein 1.

## Abbreviations

ApoB: apolipoprotein B
AW: adipose weight
BMAL1: brain and muscle ARNT-like 1
BMI: body mass index
BW: body weight
CLOCK: circadian locomotor output cycles kaput
HFD: high fat diet
KO: *Ppara-*null
LDL-C: low density lipoprotein cholesterol
LW: liver weight
PPARα: peroxisome proliferator-activated receptor α
Q-PCR: quantitative polymerase chain reaction
TC: total cholesterol
TG: triglyceride
T2DM: type 2 diabetes mellitus
WT: wild-type
ZT0: Zeitgeber 0
ZT12: Zeitgeber 12
